# Protein engineering to overcome limitations of key cytokines in cancer immunotherapy: current approaches and future perspectives

**DOI:** 10.1016/j.iotech.2025.101064

**Published:** 2025-06-21

**Authors:** U. Salazar, P. Cioffi, B. Taskoparan, I. Moraga, S. Mitra, J. vom Berg

**Affiliations:** 1 Institute of Laboratory Animal Science, University of Zurich, Schlieren, Switzerland; 2 Université de Lille, CNRS, Inserm, CHU Lille, UMR9020-U1277 CANTHER, Lille, France; 3 Division of Cell Signalling and Immunology, School of Life Sciences, University of Dundee, Dundee, UK; 4 InCephalo AG, Allschwil, Switzerland

**Keywords:** immuno-oncology, cytokine, interleukin, local administration, protein engineering, intratumoral

## Abstract

Given their central role in immune regulation, cytokines have long been considered attractive therapeutic agents, particularly in cancer immunotherapy. Despite a strong preclinical and clinical rationale, only a limited number of cytokines have been approved for cancer immunotherapy to date, and their clinical use often remains limited to specialized centers. Here we briefly review the biological traits that make some of the most widely studied cytokines—specifically, interleukin (IL)-2, IL-15, and IL-12—attractive for immunotherapy and, conversely, the challenges encountered during their clinical translation. Focusing on these three cytokines in the context of systemic or local delivery, we highlight protein engineering strategies that address challenges to increase their therapeutic index, such as poor tolerability, short serum half-life, and pleiotropy. For systemic delivery, these strategies include the use of shielded cytokines and immunocytokines to elicit tissue context-dependent activity by taking advantage of unique characteristics of the tumor microenvironment (TME). Half-life extension domains to increase serum prevalence, partial agonism to restrict activity to intended effector cells, and *cis*-targeting are also discussed. For local administration, we review protein modifications intended to increase prevalence in the tumor, including increased size, adhesion to the extracellular matrix, targeting tumor-associated antigens, or targeting immune effector cells in the TME. Looking ahead, we anticipate the development of novel approaches such as reversible, context-dependent switches, and an increasing number of combinations of individual modifications.

## Highlights

• Despite early (1980s) approvals of cytokines (e.g. IL-2, interferon α), cytokine therapy has yet to deliver on its promise.
• We review the rationale and limitations based on clinical experience and introduce protein engineering techniques to overcome them.
• Leveraging distinct features of the TME, various approaches cater to systemic or local intratumoral delivery.
• We focus on IL-2, IL-12, and IL-15 to highlight important engineering approaches for these delivery routes.
• A timeline puts the evolution of protein engineering for cytokines in temporal perspective.

## Cytokines as anticancer compounds

Cytokines are small, secreted proteins that serve as master regulators of immune responses through local cell-to-cell communication. Primarily secreted by immune cells in response to infections, inflammation, or endogenous stimuli, they play crucial roles in host defense and cancer immune surveillance. Upon binding to their multi-subunit receptors on target cells, cytokines trigger intracellular signaling cascades that alter gene transcription, ultimately orchestrating immune cell proliferation, differentiation, and activation.^1^^,^^2^ These properties have raised substantial interest in using exogenous cytokines to trigger or enhance immune responses against tumors.

**Figure 1:**
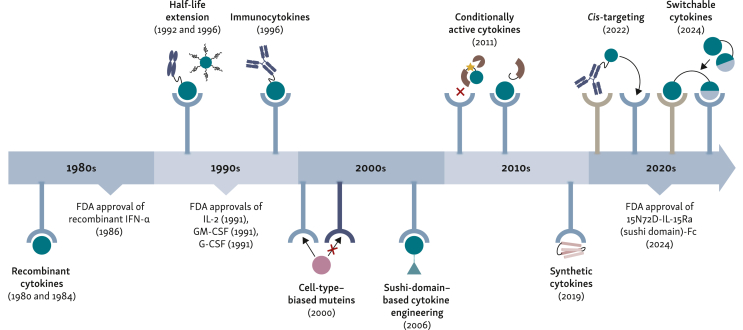
**Cytokines as anticancer compounds: from recombinant cytokines to context-dependent smart drugs.** Timeline of key advancement in cytokine engineering, illustrating the progression from the use of recombinant cytokines to advanced modification techniques. Early efforts focused on recombinant cytokine production^13^^,^^14^ and the Food and Drug Administration (FDA) approval of recombinant interferon α (IFN-α)^9^ and interleukin (IL)-2^6^^,^^7^; followed by half-life extension by Fc fusion,^15^ and PEGylation,^16^ as well as the first use of immunocytokines^17^ in the mid 1990s. Across the 2000s engineering techniques such as cell-type-biased cytokine agonists,^18^ sushi-domain-based cytokines,^19^ and conditionally active cytokines^20^ were developed. In recent years, breakthroughs in molecular engineering including synthetic cytokines,^21^*cis*-targeting fusion proteins,^22^ and switchable cytokines^23^ have enabled unprecedented precision in cytokine function and delivery with the latest FDA approval of IL-15N72D in 2023.^8^ G-CFS, granulocyte colony-stimulating factor; GM-CFS, granulocyte-macrophage colony-stimulating factor; Fc, fragment crystallizable; PEG, polyethylene glycol.

Although cytokines naturally function as local paracrine mediators, their therapeutic application typically requires systemic administration. This mismatch between physiological and therapeutic delivery, combined with their pleiotropic effects and short serum half-life,^3^, ^4^, ^5^ has posed significant challenges for clinical development. Some cytokines have been approved despite these challenges and there is considerable clinical experience in their usage^6^, ^7^, ^8^, ^9^; however, others have not reached marketing authorization due to the aforementioned limitations (reviewed by Deckers et al.^10^). To overcome these challenges and leverage the full therapeutic potential of cytokine therapies for cancer immunotherapy, a number of approaches have been explored, including local gene therapy using DNA, mRNA, or viral vectors; liposome- or nanoparticle-based formulations; and conjugation to other biomolecules.^11^^,^^12^ Here, we focus on modifications of endogenous cytokines via protein engineering approaches to cater to systemic and local intratumoral (i.t.) administration routes. Figure 1 puts these developments into a temporal perspective. We provide an overview of the biological characteristics that make three extensively studied cytokines, interleukin (IL)-2, IL-15, and IL-12, attractive for immunotherapy and discuss the challenges encountered during their clinical translation.

## Clinical experience highlights the promise and limitations of using cytokines in cancer immunotherapy

Most cytokines are broadly categorized as pro- or anti-inflammatory based on their primary roles in the immune system. Research into antitumor cytokines has historically focused on proinflammatory molecules, aiming to activate the immune system and trigger it to attack tumors that may otherwise escape its notice. Several proinflammatory cytokines, including IL-2, IL-12, and IL-15, have demonstrated considerable potential in enhancing immune responses to eliminate cancer cells.^6^^,^^7^^,^^24^, ^25^, ^26^ However, systemic toxicity is a significant challenge for these treatments (Table 1). Endogenous cytokines are tightly regulated messengers that play multiple, overlapping, context-specific roles in the immune system, and even so-called ‘proinflammatory’ cytokines can be used to both trigger and suppress immune responses.^39^, ^40^, ^41^ Addition of exogenous cytokines can unbalance this carefully maintained homeostasis, leading to systemic toxicity.

**Table 1:** Selected studies testing unmodified cytokines highlighting their promise and shortcomings in immuno-oncology

Cytokine	Clinical indication	Route of administration	Outcome	Observed positive effects	Adverse effect observed	Systemic half-life
IL-2	Renal cell carcinoma (RCC)^6^ and metastatic melanoma.^7^	Systemic i.v.	In RCC the objective response rate is 14% (5% CR and 9% PR).^6^ In metastatic melanoma the objective response rate is 16% (6% CR and 10% PR).^7^	Activation and expansion of T cells and NK cells.^27^	Activation of Tregs.^28^ Gastrointestinal: nausea, vomiting, and diarrhea in most patients.^29^^,^^30^ Cardiovascular: arrhythmias, tachycardia, arterial fibrillation.^29^ Nephrologic: azotemia, oliguria, hypotension.^29^ Dermatologic: erythematous rash, pruritus, desquamation, stomatitis.^29^ Neurologic: behavioral changes, cognitive changes, disorientation, delusion, and hallucinations.^31^ Vascular leak syndrome.^32^ In RCC and metastatic melanoma, 4% and 2% (respectively) of patients died of severe acute toxicities related to treatment.^6^^,^^7^	5-7 min^4^
IL-12	Metastatic melanoma,^33^ RCC,^34^ relapsed and refractory non-Hodgkin’s lymphoma (NHL) and Hodgkin’s disease (HD),^24^ cutaneous T-cell lymphoma (CTCL),^35^ head and neck squamous cell carcinoma.^36^^,^^37^	Systemic i.v. or s.c.^24^ intralesional in CTCL^35^; i.t. in head and neck squamous carcinoma.^36^^,^^37^	NHL 21% PR or CR. No remission in HD. Stable disease in 34% of NHL patients and 50% of HD patients.^24^ Regression of lesion in 30% melanoma patients.^33^ Stable disease in 68% of RCC patients.^34^ 56% of CTCL responded.^35^	Th1-type immunity. Stimulation of IFNγ production and cytostatic mediators by T and NK cells.^33^^,^^38^	Influenza-like symptoms: fever, fatigue, headache.^24^^,^^33^^,^^35^ Gastrointestinal: nausea, vomiting, sinusitis.^24^ Hepatic toxicity.^24^ Pulmonary toxicity.^34^ Hematologic: anemia, leukopenia, neutropenia, thrombocytopenia.^24^ Metabolic: increased transaminase and triglyceridemia.^33^^,^^34^ Dermatologic: stomatitis.^24^	5.3-9.6 h^3^
IL-15	Refractory solid tumor cancer including metastatic melanoma, RCC, non-small-cell lung cancer (NSCLC), and squamous-cell head and neck carcinoma (SCCH&N).^25^^,^^26^	Systemic i.v.^26^ or s.c.^25^	s.c. well tolerated. No objective response. Stable disease in 37% of NSCLC and RCC patients.^25^ i.v. treatment led to disease control rate of 63% of patients with advanced metastatic tumors.^26^	Mild increase of CD8+ T cells and substantial increase in NK cells, especially CD56^bright^ subset.^25^^,^^26^	Gastrointestinal: nausea and vomiting, diarrhea. Dermatologic: irritation at the site of injection. Influenza-like symptoms: fever, chills, fatigue. Cardiovascular: decreased blood pressure. Hematologic: anemia, thrombocytopenia. Metabolic: hypophosphatemia, and hypoalbuminemia. Serious events including pancreatitis, papilledema, uveitis, pneumonitis, and duodenal hemorrhage, which led to two patient deaths, were observed in intravenously treated patients.^25^^,^^26^	2.4-2.7 h^5^

CR, complete response; IFNγ, interferon γ; i.t., intratumoral; i.v., intravenous; NK, natural killer cells; PR, partial response; rhIL-12, recombinant human interleukin-12; s.c., subcutaneous; Th1, type 1 helper T cells; Tregs, regulatory T cells.

Conceptually, there are two major causes for such systemic toxicity: (i) the right effect in the wrong place—on-target, off-tumor toxicity—and (ii) the wrong effect in the wrong place—off-target toxicity.^42^ Although on-target, off-tumor toxicity can be addressed by i.t. administration and modifications that increase local retention, off-target toxicity may require strategies that address cytokine pleiotropy to restrict activity to the intended target tissue and cell type. Cytokine pleiotropy (defined as the ability of a single cytokine to elicit multiple and occasionally opposing effects in different cell types) is attributable to the expression of cytokine receptor complexes on various cell populations, resulting in context-dependent responses. The activation of multiple cell types outside the tumor, particularly in nontarget tissues, can thus lead to toxic effects outside the tumor microenvironment (TME).

Clinical experience with the widely studied interleukins IL-2, -12, and -15 provides a particularly clear illustration of the benefits and challenges of anticancer therapies based on exogenous proinflammatory cytokines. IL-2 stimulates tumor-specific cytotoxic cells, including natural killer (NK) cells and cytotoxic T lymphocytes.^27^ Research has shown IL-2 to be effective against certain cancers, and it was approved by the Food and Drug Administration (FDA) for the treatment of metastatic melanoma and renal cell carcinoma.^6^^,^^7^ However, the therapeutic efficacy of IL-2 has been limited by its pleiotropic properties: in addition to its proinflammatory targets, IL-2 also stimulates immunosuppressive regulatory T cells (Tregs), which counteract the antitumor response of effector T cells and NK cells.^39^^,^^40^ Furthermore, IL-2 treatment is associated with several toxic side effects including cardiovascular, kidney, gastrointestinal, endocrinologic, metabolic, dermatologic, and neurologic toxicities.^29^^,^^31^^,^^32^ Finally, one of the most significant toxicities associated with IL-2 treatment is an increase in vascular permeability, which can lead to severe vascular leak syndrome.^32^ This adverse event is caused by an off-target effect, since pulmonary endothelial cells express the trimeric high-affinity IL-2 receptor.^43^ Direct binding of IL-2 to endothelial cells disrupts adherent junctions and causes cytoskeletal reorganization, leading to increase permeability.^44^

Like IL-2—with which it shares receptor components—IL-15 also stimulates cytotoxic T lymphocytes and NK cells,^25^^,^^26^ and it has therefore been extensively investigated as an antitumor therapy.^45^ At moderate efficacy, systemically administered recombinant IL-15 has been linked to influenza-like symptoms such as fever, chills, and nausea. Furthermore, low blood pressure and severe side effects were documented, including duodenal hemorrhage (Table 1)^25^^,^^26^ and it has not reached marketing authorization. N-803 (brand name Anktiva), on the other hand, is an IL-15-based immunostimulatory fusion protein, comprising a modified IL-15 (N72D) with IL-15Rα sushi domain and γ receptor (FcγR) silenced IgG1Fc, which was designed for local administration. This design provides enhanced IL-2/15Rβ receptor binding, superior NK and CD8+ T cell activation, and extended half-life compared to unmodified IL-15.^46^, ^47^, ^48^ N-803 was recently approved for the local treatment of bladder cancer together with Bacillus Calmette-Guérin (BCG). In this setting, N-803 elicited a high proportion of durable complete responses.^8^

Finally, IL-12 plays a crucial role in antitumor immunity by bridging innate and adaptive immune responses. It is primarily produced by dendritic cells and macrophages in response to danger signals. IL-12 is a master regulator of type 1 immunity and is crucial for the differentiation of naive T cells into type 1 helper T cells, which are pivotal in orchestrating cell-mediated immune responses against cancer.^49^ IL-12 enhances the cytotoxic activity of T and NK cells by stimulating their production of interferon-gamma and cytotoxic mediators, enabling them to effectively target and destroy tumor cells.^33^, ^34^, ^35^, ^36^, ^37^ However, several studies have reported severe toxicity, including gastrointestinal, hepatic, pulmonary, hematologic, and metabolic toxicities, associated with IL-12 administration in cancer patients,^24^^,^^33^, ^34^, ^35^^,^^50^ with sometimes fatal outcomes.^38^

The adverse effects observed in patients (summarized in Table 1) have limited the use of cytokines in clinical settings or slowed the clinical translation of findings in preclinical models. However, they have also spurred the search for strategies to circumvent these toxic effects. These approaches can mainly be divided into two categories depending on the route of cytokine administration: local (i.t.) or systemic (summarized in Table 2).

**Table 2:** Insight into the clinical application and potential of engineered cytokines in oncology: highlighted approaches and examples

Concept	Examples	Route of administration	Stage	Comments
**Half-life extension**				
Fc fusion	DF6002 (Dragonfly)^51^	Systemic i.v.	Ph I/II NCT04423029	Monovalent IL-12 Fc fusion with FcγR silenced IgG1^51^
PEGylation	THOR-707 (Synthorx),^52^ NKTR-214 Bempegaldesleukin (Nektar)^53^^,^^54^	Systemic i.v.	Ph I/II NCT04009681 (THOR-707) Discontinued (NKTR-214)	PEGs increase half-life of molecules (here IL-2) by increasing the size over the kidney clearance cut-off; moreover they shield the molecules from enzymatic degradation and reduce the uptake by the reticuloendothelial system^55^
Albumin-fusion	MDNA11 (Medicenna)^56^	Systemic i.v.	Ph I/II NCT05086692	MDNA11 is a modified IL-2 drug that contains two amino acid modifications that abrogate the binding with CD25; also fused to HSA to increase half-life^56^
**Immunocytokines to target to TME**				
Tumor-associated vasculature	Darleukin (Philogen)^57^, ^58^, ^59^	Systemic i.v. or local i.t.	Ph I NCT02086721	IL-2-scFv fusion targeting embryonic fibronectin splice variant^59^
Exposed DNA in necrotic core	NHS-IL2,^60^ NHS-IL-12 (Merck)^61^, ^62^, ^63^	Systemic i.v.	Ph I NCT00879866 (NHS-IL2) Ph I NCT01417546 (NHS-IL12)	While NHS-IL-12 is tested in a variety of solid tumors and combinations, NHS-IL-2 has only been tested in combination with stereotactic body radiation
Tumor surface marker	RO6874281/RG7461,^64^ RO6895882/RG7813 (Hoffmann-La Roche)^65^	Systemic i.v.	Ph II NCT03386721 (RO6874281) Ph I/II NCT02004106 (RO6895882/RG7813) Ph1 NCT02350673 (RO6895882/RG7813)	Tumor-antigen targeted IL-2v are tested as monotherapy or in combination with checkpoint inhibitors
EGFR targeting	DK210 (Deka Biosciences)^66^	Systemic s.c.	NCT05704985	DK210 is a tumor-antigen targeted IL-2/IL-10 cytokine in which proinflammatory effects are balanced by anti-inflammatory effects
***Cis*-/cell-targeting**				
CD45 targeting	IL-12-aCD45 and IL-15-aCD45^67^	Local i.t.	N/A	Only tested in preclinical models so far
CD8 targeting	AB248 (Asher Biotherapeutics)^68^^,^^69^	Systemic i.v.	Ph I NCT05653882	AB248 is an anti-CD8 antibody with an attenuated IL-2 fused to the Fc region. It is tested as monotherapy or in combination with checkpoint inhibitors (pembrolizumab)
PD-1 targeting	RG6279 (Hoffmann-La Roche),^22^ ANV600 (Anaveon),^70^ PD1-IL12,^71^ PF-07209960 (Pfizer),^72^ SOT201 (SOTIO Biotech)^73^	Systemic i.v.	Ph I NCT04303858 (RG6279) Ph I/II NCT06470763 (ANV600) Ph I NCT04628780 (PF-07209960) Ph I NCT06163391 (SOT201)	PD-1 targeting compounds are tested either as monotherapy or in combination with checkpoint inhibitors
**Muteins**				
*De novo* design	NL-201 (Neurogene)^21^	Systemic i.v.	Discontinued	NL201 is a thermostable synthetic IL-2/IL-15 mimetic designed to selectively activate IL-2Rβγ signaling
Superagonist	N-803 (ImmunityBio),^8^ MDNA11 (Medicenna),^56^ SOT101 (SOTIO Biotech)^74^	Local intrabladder (N-803); systemic i.v. (MDNA11); systemic s.c. (SOT101)	Ph II/III NCT03022825 Approved (N-803) Ph I/II NCT05086692 (MDNA11) Discontinued (SOT101)	Beta-gamma biased IL-2/IL-15 variants in solid tumors using strategic dose-escalation and expansion cohorts, as both monotherapy and in combination with checkpoint inhibitors
Affinity attenuated	AB248 (Asher Biotherapeutics)^68^^,^^69^	Systemic i.v.	Ph I NCT05653882	AB248 is an anti-CD8 antibody with an attenuated IL-2 fused to the Fc region. It is tested as monotherapy or in combination with checkpoint inhibitors (pembrolizumab)
**Protease-activated unmasking**				
Active site masking	WTX-124, WTX-330 (Werewolf Therapeutics),^75^ XTX-202, XTX-301 (Xilio Development)^76^	Systemic i.v.	Ph I NCT05479812 (WTX-124) Ph I NCT05678998 (WTX-330) Ph I/II NCT05052268 (XTX-202) Ph I/II NCT05684965 (XTX-301)	WTX-124 and WTX-330 are a masked IL-2 and IL-12, respectively, which become active once the masking domain, which consists of an anti-IL-2/IL-12 antibody is cleaved by a tumor-specific protease; XTX-202 and XTX-301 are a masked IL-2 and IL-12, respectively, which become active once the masking domain, which consists of an IL-12Rβ2 domain in XTX-301 is cleaved; both Werewolf’s and Xilio’s molecules lose their half-life extension domains (Fc) upon protease cleavage^75^^,^^76^
**Increased TME retention**				
Collagen binding	CLN-617 (Cullinan Therapeutics)^77^	Local i.t.	Ph I NCT06035744	CLN-617 is single-chain fusion protein comprising LAIR2 to enhance retention in the TME, HSA to increase half-life, and two cytokines, IL-2 and IL-12^77^
IL-12-decorated aluminum-hydroxide (alum) nanoparticles	ANK-101 (Ankyra Therapeutics)^78^^,^^79^	Local i.t.	Ph I NCT06171750	Conjugating IL-12 to alum nanoparticles increases retention in (tumor) tissue; ANK-101 is retained at the site of injection up to 28 days^80^
**Cytokine fusions to modify receptor binding**				
Receptor-domain cytokine fusions	N-803 (ImmunityBio),^8^ ALKS4230 (Mural Oncology), SOT101 (SOTIO Biotech)^74^	Local intrabladder (N-803), systemic i.v. (ALKS4230, SOT101)	Ph II/III NCT03022825 (N-803) Ph I/II NCT02799095 (ALKS4230) Discontinued (SOT101)	N-803 is a modified IL-15 superagonist fused with IL-15Rα sushi domain and a silenced IgG1; approved by the FDA in 2023 in combination with BCG for patients with bladder cancer
Anti-cytokine antibody	ANV419,^81^ ANV600 (Anaveon)^70^	Systemic i.v.	Ph I NCT04855929 (ANV419) Ph I/II NCT06470763 (ANV600)	ANV419 and ANV600 comprise an anti-IL-2 antibody which binds IL-2 masking, the IL-2Rα binding site
PEGylation	THOR-707 (Syntorx),^52^ NKTR-214 (Nektar)^53^^,^^54^	Systemic i.v.	Ph I/II NCT04009681 (THOR-707) Discontinued (NKTR-214)	PEGylation is used to avoid binding to IL-2Rα via steric hindrance

Overview of key engineered cytokines currently in clinical trials or late preclinical studies, highlighting the primary engineering concept aimed at addressing specific cytokine limitations.

BCG, Bacillus Calmette-Guérin; EGFR, epidermal growth factor receptor; HSA, human serum albumin; i.t., intratumoral; i.v., intravenous; N/A, not applicable; PEG, polyethylene glycol; Ph, phase; s.c., subcutaneous; scFv, single-chain variable fragment; TME, tumor microenvironment.

## Cytokine engineering leverages unique properties of the TME

In the context of cancer immunotherapy, cytokine engineering involves the strategic modification of cytokines to enhance their therapeutic efficacy. This includes optimizing pharmacokinetics, improving target specificity, and reducing toxicity. These approaches address the inherent limitations of native cytokines, expanding their clinical potential. It should be noted that several engineering strategies are not mutually exclusive, and there can be simultaneous modulation of multiple parameters. We will focus on techniques used in the engineering of IL-2, IL-12, and IL-15. While many of the approaches below have also been conceived and applied to other cytokines such as interferons or tumor necrosis factor family members (systematically reviewed by Holder et al.^82^), the aforementioned interleukins should serve as examples to highlight engineering approaches from preclinical studies to late clinical stages.

### Systemic administration

Systemic administration via intravenous (i.v.) or subcutaneous (s.c.) infusion provides a convenient route to ensure that a therapeutic reaches its target site. Cytokines, however, require modification to avoid systemic toxicity and to ensure exclusive activity at the target site and/or target cell type. This can be achieved by immunocytokines that either target a tumor-associated antigen (TAA) or deliver the cytokine directly to the target cell (*cis*-targeting). Another option is to engineer cytokines to be biologically active only when in proximity to the tumor, exploiting the acidic TME by obtaining pH-engineered cytokines, targeting necrotic areas of the tumor, or shielding the cytokines in the bloodstream to render them inert systemically and only active once they reach the tumor. If administered systemically, the first aspect to consider for most cytokines is their short half-lives in circulation due to rapid renal clearance given their small molecular size (Table 1).

#### Half-life extension for systemic administration of cytokines

**Figure 2:**
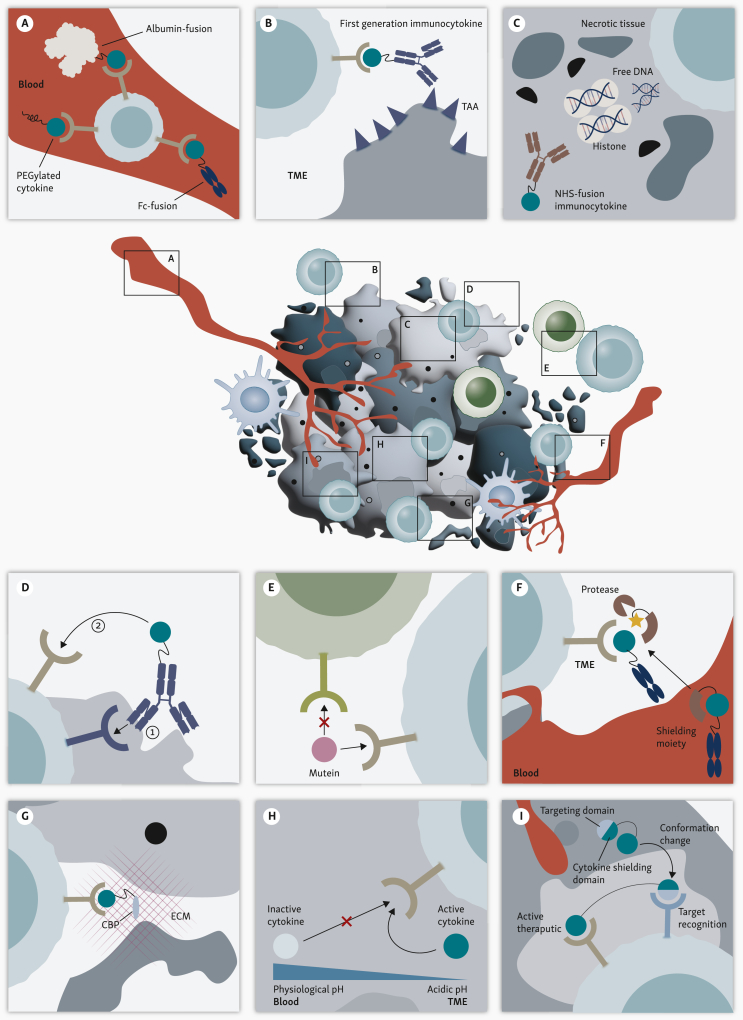
**Exploitable features of the tumor microenvironment (TME) for cytokine engineering strategies.** (A) The circulation half-life of cytokines can be extended by Fc fusion, albumin-fusion, or PEGylation. (B) Tumor-associated-antigens (TAAs) are targeted by immunocytokines, which deliver the therapeutic agent to the TME. (C) Necrotic tissues present within the tumor exhibit free DNA and histones that are targeted by NHS76-antibody fusion proteins that deliver cytokines in the TME. (D) Direct targeting of cytokines to effector cells can be achieved by first targeting a specific antigen expressed on the target cells (1), such as programmed cell death (PD)-1. Subsequently, the cytokine is delivered *in cis* (2). (E) Muteins are modified cytokines with increased or decreased affinity for specific receptor conformations, or abrogated binding to a specific receptor, compared with the unmodified cytokine. (F) Proteases present in the TME can be exploited by shielding the cytokine, rendering it inert in the circulation. Once it reaches the TME, the shielding is cleaved by proteases, releasing the active cytokine. (G) Locally administered cytokines can be fused to proteins that bind to extracellular matrix (ECM) components [i.e. collagen-binding protein (CBP)], increasing retention inside the tumor. (H) pH-sensitive cytokines are inactive at physiological pH and are activated by the acidic pH of the TME. (I) Cytokines can be designed to possess a conditional reversible activation. These modified cytokines are shielded in the absence of a sensor by a dual-specific antibody. Upon binding to its target, the antibody triggers a conformational change that frees the cytokine, enabling it to bind in the TME. Fc, fragment crystallizable; PEG, polyethylene glycol.

The short circulation time of cytokines is largely attributable to their low molecular weight, which frequently falls below the 45 kDa cut-off for kidney filtration.^83^ Consequently, the use of cytokines as therapeutic agents necessitates the administration of repeated doses to maintain therapeutic levels, which can be challenging in clinical practice. For example, rhIL-2 typically requires a high-frequency i.v. dosing schedule every 8 hours over a 5-day period.^7^ A common approach to extend the half-life is to fuse wild-type cytokines to the immunoglobulin fragment crystallizable (Fc) portion or to human serum albumin via a flexible peptide linker (Figure 2A). This increases molecular size above the renal filtration threshold, preventing rapid clearance through the kidneys, and exploits the neonatal Fc receptor (FcRn) recycling pathway.^84^^,^^85^ Fc fusion of cytokines may trigger unwanted Fc-mediated effector functions, such as antibody-dependent cellular cytotoxicity or complement activation, by binding to FcγR. Therefore, Fc fragments are often mutated to prevent these interactions (reviewed by Yang et al.^86^).

An alternative approach to half-life extension is polymer conjugation, particularly with polyethylene glycol (PEG). PEGylation typically involves the covalent linkage of PEG to exposed amino groups on the surface of the cytokine. PEGylated cytokines have a higher molecular weight than the renal filtration cut-off and are thus not immediately excreted. Furthermore, PEGylation offers protection against enzymatic degradation and reduces reticuloendothelial system uptake by steric hindrance.^55^^,^^87^ PEGylation was prominently used in the discontinued Nektar Therapeutics’ PEGylated IL-2 (NKTR-214), a prodrug consisting of IL-2 conjugated with six PEG molecules. NKTR-214 remains inactive in its fully PEGylated form and becomes active only when hydrolysis removes four or five PEG molecules, gradually exposing the active IL-2 molecule.^53^ In contrast, THOR-707 uses a novel method of PEGylation where the PEG moiety is site specifically and irreversibly bound via click chemistry.^52^ THOR-707 is currently undergoing a phase I/II clinical trial and was reported to have a half-life of ∼10 hours (NCT04009681).

Half-life extension modifications are an integral part of the design of cytokine constructs intended for systemic administration. However, while improving time-on-target, half-life extension is neither increasing tolerability nor a sufficient strategy to guarantee efficacy against tumors. In the upcoming sections, we will discuss engineering strategies aimed at increasing on-target efficacy and reducing the toxic effects of systemically delivered cytokines.

#### Immunocytokines—cytokines with a homing moiety

First-generation immunocytokines, which link cytokines to antibodies targeting TAAs such as extra domain B fibronectin splice variants, carcinoembryonic antigen-related cell adhesion molecule, or fibroblast activation protein, have demonstrated the potential to enhance the localization and accumulation and thus the effectiveness of cancer therapy (Figure 2B).^57^^,^^64^^,^^65^ However, this targeting is less effective with systemic administration, where the distribution of the fusion protein is influenced by the expression of cognate cytokine receptors. This limitation highlights the inherently bimodal properties of immunocytokines, with both the antibody and the cytokine capable of anchoring the therapeutic agent by binding to the TAA or to the cytokine’s endogenous receptor, respectively. A careful balancing of affinities is needed between the antibody and the cytokine. If the affinity toward the cytokine receptor exceeds that for the TAA, a systemic ‘sink’ effect might result from NK and NKT cells, which would divert the cytokines away from the intended intratumoral CD8^+^ T cells.^88^ This phenomenon may help explain the limited clinical success of first-generation immunocytokines.

More innovative approaches consist of delivering to TAAs dual cytokines, which comprise different cytokines with similar but also opposite effects. DK210 is a dual cytokine which is composed of IL-2 and IL-10 cytokines fused to an anti-epidermal growth factor receptor targeting moiety. DK210 mitigates the toxicity associated with IL-2 treatment by the anti-inflammatory effects of IL-10.^66^ Currently, DK210 is being evaluated in a phase I clinical trial (NCT05704985).

Other immunocytokine designs target free DNA and histones exposed in the necrotic areas of tumors (Figure 2C). One example is IL-12 fused with NHS76, an antibody that binds specifically to DNA/histone complexes. When administered s.c., IL-12-NHS has demonstrated significant antitumor activity in syngeneic murine models.^89^ Currently, this approach is under evaluation in a phase I clinical trial for patients with metastatic solid tumors.^61^, ^62^, ^63^

Although IL-2 and IL-12 immunocytokines targeting TAAs or TME necrotic regions have been extensively investigated, further engineering techniques such as *cis*-targeting and affinity modulation (discussed in the following sections) could enhance their effectiveness in solid tumors.

#### *Cis*-targeting immunocytokines

Another strategy for reducing the toxic and adverse pleiotropic effects of systemically administered cytokines is to direct the cytokine specifically to tumor-specific immune cells by targeting surface antigens that are expressed by those cells. Programmed cell death protein 1 (PD-1) is a commonly targeted marker in antitumor therapies, as it is one of the first membrane proteins expressed by immune cells (especially CD4+ and CD8+ T cells) after antigen-(re)encounter. The cytokine fusion protein binds to the immune cells expressing the marker, effectively delivering the cytokine *in cis* (Figure 2D). A critical factor is achieving the right ‘balance’ between the affinity of the antibody delivering the cytokine and the cytokine’s interaction with its receptor. To minimize off-target effects and to ensure delivery to the target cells, the antibody’s affinity for its target should be in the range of the cytokine for its receptor.^90^

One example of a *cis*-targeted cytokine fusion protein is PD1-IL2v, which comprises an IL-2 variant fused to the C-terminus of a blocking anti-PD-1 antibody.^22^ This therapeutic approach has demonstrated efficacy in a variety of syngeneic murine tumor models and currently is undergoing evaluation in a phase I clinical trial (NCT04303858). Similarly, ANV600—a PD-1-targeted anti-IL2/IL-2 bispecific antibody—demonstrated tumor-inhibiting effects in both B16F10 and MC38 tumor models^70^ and is currently being evaluated in clinical trials as a monotherapy or in combination with PD-1 blockade (NCT06470763).

Although the concept of *cis*-targeting of an attenuated cytokine is predominantly explored for IL-2, IL-12- and IL-15-based designs have used *cis*-targeting: Zou et al. developed a novel IL-12/PD-1 fusion protein containing a mutated low-affinity IL-12, which reduces toxicity by minimizing excessive NK cell activation in circulation, while delivering IL-12 to PD-1 expressing cells *in cis*.^71^ Constructs comprising a mutated affinity-attenuated IL-15 targeted to PD-1 have also been developed and are currently being investigated in clinical trials (NCT04628780 and NCT06163391).^72^^,^^73^

In conclusion, *cis*-targeting improves the balance between efficacy and safety, however, immunocytokine accessibility to tumor-specific immune cells may be hindered by poor penetration in solid tumors.

#### Affinity modulation of cytokines

As previously stated, the specificity of *cis*-targeting depends on the antibody having a higher affinity for its antigen than the cytokine has for its receptor. This balance ensures that *cis*-targeting immunocytokines avoid a systemic ‘sink’ effect and reduces systemic toxicity. To achieve this, novel strategies are being developed to modulate the affinity of cytokines for their natural receptors.^21^^,^^71^^,^^91^^,^^92^ For instance, amino acid mutations can be introduced to increase or decrease the protein’s affinity for its receptor or specific receptor conformations. These engineered cytokine variants are known as muteins.

Pleiotropic effects of cytokines can result from their ability to bind to different receptor conformations. For example, IL-2 can interact with either the dimeric IL-2 receptor (IL-2Rβγ) or the high-affinity trimeric receptor (IL-2Rαβγ). To mitigate these pleiotropic effects, the affinity of cytokines can be enhanced toward specific receptor conformations. Novel superagonists such as NL-201 and MDNA11 are designed to optimize IL-2 signaling by enhancing IL-2Rβγ binding while avoiding IL-2Rα (CD25), thereby reducing Treg activation (Figure 2E).^21^^,^^56^ MDNA11 is currently in clinical phase I testing (NCT05086692).

Cytokine affinity modulation can also be exploited to influence the differentiation of target T cells. For instance, strong IL-2 signaling promotes effector T cell differentiation, whereas weaker signals favor memory T cell formation.^93^, ^94^, ^95^, ^96^ This underlines the importance of not only targeting specific cell types but also regulating the intensity of signaling to effectively regulate immune functions. This differential response enabled the development of high-affinity IL-2 agonists that decouple T cell proliferation from terminal differentiation for therapeutic immune modulation.^95^^,^^96^

#### Shielding

One of the more recent strategies for minimizing off-target toxicity involves the shielding of cytokines in the bloodstream using a masking domain that is fused to the cytokine via a linker containing a recognition motif for a tumor-specific protease. This allows the shielded cytokines to remain inactive until they reach the tumor site (Figure 2F). There, matrix metalloproteases that are enriched in the TME cleave off the cytokine-shielding domains and release the cytokine to act locally within the TME.^97^, ^98^, ^99^ This method reduces systemic exposure and ensures that immune activation is concentrated at the tumor site, thereby decreasing the risk of severe side effects and improving treatment specificity. For example, Wu et al. developed a PD-1-targeted, receptor-masked IL-2 immunocytokine that selectively engages IL-2Rα on PD-1-positive T cells, providing potent antitumor efficacy while minimizing systemic toxicity.^97^ This engineered cytokine retains the ability to engage IL-2Rα but is masked to attenuate its activity until it reaches the tumor site, thus significantly reducing the side effects often associated with high-dose IL-2 therapies.^97^ A similar approach was used to shield IL-15 and IL-2 until they reached the tumor site, where tumor-specific proteases subsequently rendered the cytokine active.^98^^,^^99^

Shielding moieties are often combined with half-life extension modifications. For example, in the Xilio platform used in the cytokine fusion proteins XTX-301 and XTX-202 (engineered IL-12 and IL-2, respectively), tumor-specific proteases activate the cytokine fusion protein by cleaving the masking domain as well as the half-life extension domain.^76^ Similarly, the Werewolf platform (WTX-330 and WTX-124) cleaves both the half-life and the masking domains, leaving only the active cytokine.^75^ This strategy ensures that the activated cytokine is swiftly degraded in case it leaks from the TME into the bloodstream.

### Local administration

Most cytokines have evolved as autocrine or paracrine molecules that exert their effects at the site of release. Local delivery of cytokines by i.t. administration mimics this physiologic mechanism of action and should allow for exclusive exposure of the TME to potent therapeutic agents. This approach thus promises to reduce the risk of systemic toxicity and unwanted pleiotropic effects. On the other hand, i.t. delivery is faced with the difficulty of local administration in certain cancer types^42^ and the high interstitial tumor tissue pressure that can prevent injected cytokines from reaching target cells.^100^ Locally delivered cytokines may also leak into the systemic circulation, resulting in dose-limiting systemic toxicity.^36^^,^^101^^,^^102^ To prevent this leakage, therapeutic cytokines can be modified to increase their retention in the TME upon i.t. delivery.

#### Cytokines fused to adhesion molecules

IL-12 has been the subject of extensive investigation for local administration, as systemic administration of this cytokine has been associated with significant toxic effects. To address this issue, localized i.t. delivery has emerged as a promising strategy, allowing effective concentration of IL-12 within the tumor while avoiding systemic exposure to high concentrations.^11^ To reduce the leakage of IL-12 into the systemic circulation, IL-12 was anchored within the TME by conjugating the cytokine with collagen-binding proteins (CBPs) such as lumican or leukocyte-associated immunoglobulin-like receptor 2 (LAIR2), resulting in minimal peripheral exposure while maintaining effective concentration in the TME (Figure 2G).^77^^,^^103^ CBPs have also been used for the local administration of cytokine combinations. CLN-617, a single-chain fusion protein comprising IL-2, LAIR2, human serum albumin, and IL-12, triggered robust immune-mediated tumor destruction in preclinical models while maintaining cytokine concentrations at the tumor site.^77^ It is currently being tested in the clinic (NCT06035744). Cytokine combinations like CLN-617 leverage multiple immune pathways to enhance the overall antitumor response. To avoid sequestration of such dual cytokine constructs to only one receptor, affinity adjustments in some formats may be required.

#### Increasing size of cytokines

Preclinical work testing LAIR-mouse serum albumin-IL-2 fusions with varying combinations and LAIR collagen affinities has demonstrated that, next to adhesion to extracellular matrix components in the TME, the size of a cytokine fusion construct may play an at least similarly important role in tumor retention upon local delivery.^104^ Conjugating cytokines to aluminum-hydroxide (alum) nanoparticles, a commonly utilized adjuvant in vaccines, results in a dramatic size increase. Alum binds to phosphorylated, engineered peptide tags on cytokines such as IL-12 or IL-2.^78^ These alum-conjugated nanoparticles can remain at the injection site for weeks, providing sustained release without systemic toxicity.^78^ A recent example of this approach is ANK-101, which is currently undergoing clinical investigation (NCT06171750).^79^

#### Locally applied immunocytokines

Another strategy is to combine a cytokine with a classical binder moiety targeting unique antigens in the TME. Although the intended administration route for most immunocytokines is i.v. or s.c., the binder/homing moiety may also facilitate tissue retention upon local delivery.^58^ A recent study by Santollani et al. introduced an interesting variant of i.t. delivery by using cell surface targeting to immune effector cells rather than unique tumor-associated structures.^67^ In murine flank tumor models such as MC38 and B16F10, the combination of anti-CD45-IL12 followed by anti-CD45-IL15 i.t. injections resulted in the complete eradication of tumors with no toxic effects observed.^67^

Cytokines can also be engineered for an increased therapeutic window for local treatment of a particular organ or tissue: Beffinger and colleagues recently described an FcRn-silenced IL-12Fc fusion cytokine designed for local brain cancer therapy. It demonstrated high brain retention and efficacy yet minimal systemic exposure,^105^ as both FcRn-mediated brain-to-blood transcytosis and peripheral recycling are abolished,^102^ while the substantially larger size ensures prolonged tissue retention.

In conclusion, i.t. administration of engineered cytokines can provide a powerful and targeted approach to cancer treatment, localizing therapeutic effects within the tumor while minimizing systemic toxicity. Despite this localized effect, i.t. cytokine administration has the potential to elicit abscopal effects. This phenomenon occurs when local treatment of a tumor initiates a systemic immune response, leading to the destruction of distant metastases or lesions that were not directly treated. In essence, the injected tumor acts as a vaccine, priming the T cells by activating antigen-presenting cells in the TME to recognize and attack cancer cells throughout the body.^42^ This immune-mediated response illustrates the potential of i.t. therapies in treating metastatic cancers and is more attainable due to concentrated delivery at the tumor site. In contrast to systemic toxicity, which arises from off-target effects and can harm healthy tissues, the abscopal effect is a most welcome ‘side-effect’ of i.t. treatment.

### New avenues: conditional, reversible activity

While currently in preclinical stage, two recent concepts explore another layer of tumor-selective activity, as they not only allow TME-dependent activation of an otherwise inert cytokine moiety but also resume the inactive state in case of leakage from the tumor bed.

#### pH-engineered cytokines

Tumor environments typically exhibit low pH levels due to altered metabolic processes such as increased glycolysis,^106^ which presents an opportunity to develop therapeutics, including engineered cytokines, that are activated by acidic conditions (Figure 2H).^107^ This strategy aims to ensure that immune activation occurs primarily within the acidic TME, reducing systemic toxicity. However, while pH-engineered IL-2 can accumulate in tumors, its receptor binding and activity may still be compromised by the acidic tumor environment. Addressing this, Gaggero et al. developed Switch-2, an engineered IL-2 variant that shows enhanced IL-2Rα binding, which triggers preferential signaling in acidic versus neutral pH, achieving high antitumoral activity.^108^ Currently in preclinical state, future work will need to address the translatability to humans and its tumor selectivity and the broader applicability of this approach to a variety of tumor sizes and metabolic states.

#### Allosteric switches

A relatively novel approach uses antibodies engineered to bind two antigens with varying affinities, allowing the development of a new class of therapeutics outfitted with ‘sensors’ with localized conditional activity after being systemically administered. In such designs, the dual-binding antibody shields a cytokine (e.g. IL-2) unless it encounters its target with high-affinity (e.g. LAG-3) on antigen-experienced effector cells.^23^ This dual antibody shielding/sensor concept is applicable to other cytokines and TME markers such as IL-12 and PD-1, PD-L1, and extracellular ATP, respectively. Such an approach should also ensure that the therapeutic becomes inactive again if it dissociates from the intended *cis*-target or TME marker (Figure 2I).

## Conclusions

While immunotherapeutic approaches such as immune checkpoint blockade are part of the standard of care for an increasing number of indications, cytokines and many agonistic antibodies have not yet fully delivered on their therapeutic potential. An increasing number of engineering approaches to overcome the shortcomings of first-generation cytokine drugs, tailored to systemic or local administration, are now being tested in the clinic. As our knowledge increases on the clinical impact of individual strategies, it is likely that a combination of existing modifications, including multiple-cytokine combinations, will eventually lead to new dynamics in the cytokine field. Future strategies will likely comprise reversible on-demand activity via conditional activation, target organ-specific designs beyond the TME, and combinations of formulation and protein engineering. Controlled release formulations that mimic natural cytokine dynamics and combination therapies to balance stimulatory and regulatory signals can add additional benefit to engineered cytokines. Of note, gene therapy approaches to express modified cytokines rather than endogenous variants may further advance cytokine therapy of cancer. Finally, many more cytokines including IL-21, tumor necrosis factor α, interferon α, and IL-18, to name a few, have been the focus of elegant engineering approaches and are in some cases close to approval.

## References

[1] Briukhovetska D., Dorr J., Endres S., Libby P., Dinarello C.A., Kobold S. (2021). Interleukins in cancer: from biology to therapy. Nat Rev Cancer.

[2] Stark G.R., Darnell J.E. (2012). The JAK-STAT pathway at twenty. Immunity.

[3] Atkins M.B., Robertson M.J., Gordon M. (1997). Phase I evaluation of intravenous recombinant human interleukin 12 in patients with advanced malignancies. Clin Cancer Res.

[4] Lotze M.T., Frana L.W., Sharrow S.O., Robb R.J., Rosenberg S.A. (1985). In vivo administration of purified human interleukin 2. I. Half-life and immunologic effects of the Jurkat cell line-derived interleukin 2. J Immunol.

[5] Conlon K.C., Lugli E., Welles H.C. (2015). Redistribution, hyperproliferation, activation of natural killer cells and CD8 T cells, and cytokine production during first-in-human clinical trial of recombinant human interleukin-15 in patients with cancer. J Clin Oncol.

[6] Fyfe G., Fisher R.I., Rosenberg S.A., Sznol M., Parkinson D.R., Louie A.C. (1995). Results of treatment of 255 patients with metastatic renal cell carcinoma who received high-dose recombinant interleukin-2 therapy. J Clin Oncol.

[7] Atkins M.B., Lotze M.T., Dutcher J.P. (1999). High-dose recombinant interleukin 2 therapy for patients with metastatic melanoma: analysis of 270 patients treated between 1985 and 1993. J Clin Oncol.

[8] Chamie K., Chang S.S., Kramolowsky E.V. (2024). Quality of life in the phase 2/3 trial of N-803 plus bacillus Calmette-Guerin in bacillus Calmette-Guerin‒unresponsive nonmuscle-invasive bladder cancer. Urol Pract.

[9] Kirkwood J.M., Strawderman M.H., Ernstoff M.S., Smith T.J., Borden E.C., Blum R.H. (1996). Interferon alfa-2b adjuvant therapy of high-risk resected cutaneous melanoma: the Eastern Cooperative Oncology Group Trial EST 1684. J Clin Oncol.

[10] Deckers J., Anbergen T., Hokke A.M. (2023). Engineering cytokine therapeutics. Nat Rev Bioeng.

[11] Nguyen K.G., Vrabel M.R., Mantooth S.M. (2020). Localized interleukin-12 for cancer immunotherapy. Front Immunol.

[12] Kwong B., Gai S.A., Elkhader J., Wittrup K.D., Irvine D.J. (2013). Localized immunotherapy via liposome-anchored anti-CD137 + IL-2 prevents lethal toxicity and elicits local and systemic antitumor immunity. Cancer Res.

[13] Rosenberg S.A., Grimm E.A., McGrogan M. (1984). Biological activity of recombinant human interleukin-2 produced in Escherichia coli. Science.

[14] Goeddel D.V., Yelverton E., Ullrich A. (1980). Human leukocyte interferon produced by E. coli is biologically active. Nature.

[15] Harvill E.T., Fleming J.M., Morrison S.L. (1996). In vivo properties of an IgG3-IL-2 fusion protein. A general strategy for immune potentiation. J Immunol.

[16] Rosen P., Karasiewicz R., Nalin C. (1995).

[17] Becker J.C., Varki N., Gillies S.D., Furukawa K., Reisfeld R.A. (1996). An antibody-interleukin 2 fusion protein overcomes tumor heterogeneity by induction of a cellular immune response. Proc Natl Acad Sci U S A.

[18] Shanafelt A.B., Lin Y., Shanafelt M.C. (2000). A T-cell-selective interleukin 2 mutein exhibits potent antitumor activity and is well tolerated in vivo. Nat Biotechnol.

[19] Mortier E., Quemener A., Vusio P. (2006). Soluble interleukin-15 receptor alpha (IL-15R alpha)-sushi as a selective and potent agonist of IL-15 action through IL-15R beta/gamma. Hyperagonist IL-15 x IL-15R alpha fusion proteins. J Biol Chem.

[20] Puskas J., Skrombolas D., Sedlacek A., Lord E., Sullivan M., Frelinger J. (2011). Development of an attenuated interleukin-2 fusion protein that can be activated by tumour-expressed proteases. Immunology.

[21] Silva D.A., Yu S., Ulge U.Y. (2019). De novo design of potent and selective mimics of IL-2 and IL-15. Nature.

[22] Codarri Deak L., Nicolini V., Hashimoto M. (2022). PD-1-cis IL-2R agonism yields better effectors from stem-like CD8(+) T cells. Nature.

[23] Killebrew J., Okada S., Amon L. (2024). Abstract 4062: A novel method for generating regulated cytokine therapeutics: safety and activity of a conditionally active cLAG3-IL2 capable of delivering IL2 to LAG3+ cells while remaining inert on LAG3- cells. Cancer Res.

[24] Younes A., Pro B., Robertson M.J. (2004). Phase II clinical trial of interleukin-12 in patients with relapsed and refractory non-Hodgkin’s lymphoma and Hodgkin’s disease. Clin Cancer Res.

[25] Miller J.S., Morishima C., McNeel D.G. (2018). A first-in-human phase I study of subcutaneous outpatient recombinant human IL15 (rhIL15) in adults with advanced solid tumors. Clin Cancer Res.

[26] Conlon K.C., Potter E.L., Pittaluga S. (2019). IL15 by continuous intravenous infusion to adult patients with solid tumors in a phase I trial induced dramatic NK-cell subset expansion. Clin Cancer Res.

[27] Hefeneider S.H., Conlon P.J., Henney C.S., Gillis S. (1983). In vivo interleukin 2 administration augments the generation of alloreactive cytolytic T lymphocytes and resident natural killer cells. J Immunol.

[28] Almeida A.R., Legrand N., Papiernik M., Freitas A.A. (2002). Homeostasis of peripheral CD4+ T cells: IL-2R alpha and IL-2 shape a population of regulatory cells that controls CD4+ T cell numbers. J Immunol.

[29] Margolin K.A., Rayner A.A., Hawkins M.J. (1989). Interleukin-2 and lymphokine-activated killer cell therapy of solid tumors: analysis of toxicity and management guidelines. J Clin Oncol.

[30] Rosenberg S.A., Lotze M.T., Yang J.C. (1989). Experience with the use of high-dose interleukin-2 in the treatment of 652 cancer patients. Ann Surg.

[31] Denicoff K.D., Rubinow D.R., Papa M.Z. (1987). The neuropsychiatric effects of treatment with interleukin-2 and lymphokine-activated killer cells. Ann Intern Med.

[32] Siegel J.P., Puri R.K. (1991). Interleukin-2 toxicity. J Clin Oncol.

[33] Bajetta E., Del Vecchio M., Mortarini R. (1998). Pilot study of subcutaneous recombinant human interleukin 12 in metastatic melanoma. Clin Cancer Res.

[34] Motzer R.J., Rakhit A., Schwartz L.H. (1998). Phase I trial of subcutaneous recombinant human interleukin-12 in patients with advanced renal cell carcinoma. Clin Cancer Res.

[35] Rook A.H., Wood G.S., Yoo E.K. (1999). Interleukin-12 therapy of cutaneous T-cell lymphoma induces lesion regression and cytotoxic T-cell responses. Blood.

[36] van Herpen C.M., Looman M., Zonneveld M. (2004). Intratumoral administration of recombinant human interleukin 12 in head and neck squamous cell carcinoma patients elicits a T-helper 1 profile in the locoregional lymph nodes. Clin Cancer Res.

[37] van Herpen C.M., van der Laak J.A., de Vries I.J. (2005). Intratumoral recombinant human interleukin-12 administration in head and neck squamous cell carcinoma patients modifies locoregional lymph node architecture and induces natural killer cell infiltration in the primary tumor. Clin Cancer Res.

[38] Leonard J.P., Sherman M.L., Fisher G.L. (1997). Effects of single-dose interleukin-12 exposure on interleukin-12-associated toxicity and interferon-gamma production. Blood.

[39] Antony P.A., Restifo N.P. (2005). CD4+CD25+ T regulatory cells, immunotherapy of cancer, and interleukin-2. J Immunother.

[40] Sitrin J., Ring A., Garcia K.C., Benoist C., Mathis D. (2013). Regulatory T cells control NK cells in an insulitic lesion by depriving them of IL-2. J Exp Med.

[41] Masli S., Turpie B. (2009). Anti-inflammatory effects of tumour necrosis factor (TNF)-alpha are mediated via TNF-R2 (p75) in tolerogenic transforming growth factor-beta-treated antigen-presenting cells. Immunology.

[42] Melero I., Castanon E., Alvarez M., Champiat S., Marabelle A. (2021). Intratumoural administration and tumour tissue targeting of cancer immunotherapies. Nat Rev Clin Oncol.

[43] Krieg C., Letourneau S., Pantaleo G., Boyman O. (2010). Improved IL-2 immunotherapy by selective stimulation of IL-2 receptors on lymphocytes and endothelial cells. Proc Natl Acad Sci U S A.

[44] Wylezinski L.S., Hawiger J. (2016). Interleukin 2 activates brain microvascular endothelial cells resulting in destabilization of adherens junctions. J Biol Chem.

[45] Waldmann T.A., Dubois S., Miljkovic M.D., Conlon K.C. (2020). IL-15 in the combination immunotherapy of cancer. Front Immunol.

[46] Kim P.S., Kwilas A.R., Xu W. (2016). IL-15 superagonist/IL-15RαSushi-Fc fusion complex (IL-15SA/IL-15RαSu-Fc; ALT-803) markedly enhances specific subpopulations of NK and memory CD8+ T cells, and mediates potent anti-tumor activity against murine breast and colon carcinomas. Oncotarget.

[47] Foltz J.A., Hess B.T., Bachanova V. (2021). Phase I trial of N-803, an IL15 receptor agonist, with rituximab in patients with indolent non-Hodgkin lymphoma. Clin Cancer Res.

[48] Rosario M., Liu B., Kong L. (2016). The IL-15-based ALT-803 complex enhances FcγRIIIa-triggered NK cell responses and in vivo clearance of B cell lymphomas. Clin Cancer Res.

[49] Tugues S., Burkhard S.H., Ohs I. (2015). New insights into IL-12-mediated tumor suppression. Cell Death Differ.

[50] Car B.D., Eng V.M., Lipman J.M., Anderson T.D. (1999). The toxicology of interleukin-12: a review. Toxicol Pathol.

[51] Gutierrez E., Bigelow M., LaCroix C. (2023). An optimized IL-12-Fc expands its therapeutic window, achieving strong activity against mouse tumors at tolerable drug doses. Med.

[52] Ptacin J.L., Caffaro C.E., Ma L. (2021). An engineered IL-2 reprogrammed for anti-tumor therapy using a semi-synthetic organism. Nat Commun.

[53] Charych D.H., Hoch U., Langowski J.L. (2016). NKTR-214, an engineered cytokine with biased IL2 receptor binding, increased tumor exposure, and marked efficacy in mouse tumor models. Clin Cancer Res.

[54] Diab A., Gogas H., Sandhu S. (2023). Bempegaldesleukin plus nivolumab in untreated advanced melanoma: the open-label, phase III PIVOT IO 001 trial results. J Clin Oncol.

[55] Lawrence P.B., Price J.L. (2016). How PEGylation influences protein conformational stability. Curr Opin Chem Biol.

[56] Merchant R., Galligan C., Munegowda M.A. (2022). Fine-tuned long-acting interleukin-2 superkine potentiates durable immune responses in mice and non-human primate. J Immunother Cancer.

[57] Weide B., Eigentler T., Catania C. (2019). A phase II study of the L19IL2 immunocytokine in combination with dacarbazine in advanced metastatic melanoma patients. Cancer Immunol Immunother.

[58] Weide B., Eigentler T.K., Pflugfelder A. (2014). Intralesional treatment of stage III metastatic melanoma patients with L19-IL2 results in sustained clinical and systemic immunologic responses. Cancer Immunol Res.

[59] Carnemolla B., Borsi L., Balza E. (2002). Enhancement of the antitumor properties of interleukin-2 by its targeted delivery to the tumor blood vessel extracellular matrix. Blood.

[60] van den Heuvel M.M., Verheij M., Boshuizen R. (2015). NHS-IL2 combined with radiotherapy: preclinical rationale and phase Ib trial results in metastatic non-small cell lung cancer following first-line chemotherapy. J Transl Med.

[61] Gatti-Mays M.E., Tschernia N.P., Strauss J. (2023). A phase I single-arm study of biweekly NHS-IL12 in patients with metastatic solid tumors. Oncologist.

[62] Strauss J., Deville J.-L., Sznol M. (2023). First-in-human phase Ib trial of M9241 (NHS-IL12) plus avelumab in patients with advanced solid tumors, including dose expansion in patients with advanced urothelial carcinoma. J Immunother Cancer.

[63] Strauss J., Heery C.R., Kim J.W. (2019). First-in-human phase I trial of a tumor-targeted cytokine (NHS-IL12) in subjects with metastatic solid tumors. Clin Cancer Res.

[64] Prenen H., Deva S., Keam B. (2024). Phase II study to determine the antitumor activity and safety of Simlukafusp alfa (FAP-IL2v) combined with atezolizumab in esophageal cancer. Clin Cancer Res.

[65] Klein C., Waldhauer I., Nicolini V.G. (2017). Cergutuzumab amunaleukin (CEA-IL2v), a CEA-targeted IL-2 variant-based immunocytokine for combination cancer immunotherapy: overcoming limitations of aldesleukin and conventional IL-2-based immunocytokines. Oncoimmunology.

[66] Ahn J.J., Dudics S., Langan D.P. (2025). Coupling IL-2 with IL-10 to mitigate toxicity and enhance antitumor immunity. Cell Rep Med.

[67] Santollani L., Maiorino L., Zhang Y.J. (2024). Local delivery of cell surface-targeted immunocytokines programs systemic antitumor immunity. Nat Immunol.

[68] Spigel D.R., Albany C., Chisamore M. (2023). 2023. 753 An open-label, phase 1a/b study of AB248, a CD8+ selective IL-2 mutein fusion protein, alone or in combination with pembrolizumab in patients with advanced solid tumors. J Immunother Cancer.

[69] Kaptein P., Slingerland N., Metoikidou C. (2024). CD8-targeted IL2 unleashes tumor-specific immunity in human cancer tissue by reviving the dysfunctional T-cell pool. Cancer Discov.

[70] Murer P., Petersen L., Egli N. (2025). ANV600 is a novel PD-1 targeted IL-2Rβγ agonist that selectively expands tumor antigen-specific T cells and potentiates PD-1 checkpoint inhibitor therapy. J Immunother Cancer.

[71] Zou Z., Shen J., Xue D. (2024). Anti-PD-1 cis-delivery of low-affinity IL-12 activates intratumoral CD8(+)T cells for systemic antitumor responses. Nat Commun.

[72] Naing A., McKean M., Rosen L.S. (2025). First-in-human phase I study to evaluate safety, tolerability, pharmacokinetics, pharmacodynamics, immunogenicity, and antitumor activity of PF-07209960 in patients with advanced or metastatic solid tumors. ESMO Open.

[73] Matuskova H., Marasek P., Mazhara V. (2025). Novel PD-1-targeted, activity-optimized IL-15 mutein SOT201 acting in cis provides antitumor activity superior to PD1-IL2v. J Immunother Cancer.

[74] Champiat S., Garralda E., Galvao V. (2025). Nanrilkefusp alfa (SOT101), an IL-15 receptor βγ superagonist, as a single agent or with anti-PD-1 in patients with advanced cancers. Cell Rep Med.

[75] Nirschl C.J., Brodkin H.R., Domonkos C. (2023). mWTX-330, an IL-12 INDUKINE molecule, activates and reshapes tumor-infiltrating CD8+ T and NK cells to generate antitumor immunity. Cancer Immunol Res.

[76] Patel E., Malkova N.V., Crowe D. (2024). XTX301, a tumor-activated interleukin-12 has the potential to widen the therapeutic index of IL12 treatment for solid tumors as evidenced by preclinical studies. Mol Cancer Ther.

[77] Mehta N.K., Rakhra K., Meetze K.A. (2024). CLN-617 retains IL2 and IL12 in injected tumors to drive robust and systemic immune-mediated antitumor activity. Cancer Immunol Res.

[78] Agarwal Y., Milling L.E., Chang J.Y.H. (2022). Intratumourally injected alum-tethered cytokines elicit potent and safer local and systemic anticancer immunity. Nat Biomed Eng.

[79] Park J.C., Curti B., Butler M.O. (2025). Abstract CT039: Results of a first-in-human phase 1 trial of anchored IL-12 drug conjugate (ANK-101). Cancer Res.

[80] Park J.C., Butler M.O., Curti B.D. (2024). A phase 1, open-label, dose escalation study on the safety and tolerability of ANK-101 in advanced solid tumors.

[81] Murer P., Brannetti B., Rondeau J.M. (2024). Discovery and development of ANV419, an IL-2/anti-IL-2 antibody fusion protein with potent CD8+ T and natural killer cell-stimulating capacity for cancer immunotherapy. MAbs.

[82] Holder P.G., Lim S.A., Huang C.S. (2022). Engineering interferons and interleukins for cancer immunotherapy. Adv Drug Deliv Rev.

[83] Anderson N.L., Anderson N.G. (2002). The human plasma proteome: history, character, and diagnostic prospects. Mol Cell Proteomics.

[84] Bern M., Sand K.M., Nilsen J., Sandlie I., Andersen J.T. (2015). The role of albumin receptors in regulation of albumin homeostasis: implications for drug delivery. J Control Release.

[85] Roopenian D.C., Christianson G.J., Sproule T.J. (2003). The MHC class I-like IgG receptor controls perinatal IgG transport, IgG homeostasis, and fate of IgG-Fc-coupled drugs. J Immunol.

[86] Yang C., Gao X., Gong R. (2017). Engineering of Fc fragments with optimized physicochemical properties implying improvement of clinical potentials for Fc-based therapeutics. Front Immunol.

[87] Li S.D., Huang L. (2009). Nanoparticles evading the reticuloendothelial system: role of the supported bilayer. Biochim Biophys Acta.

[88] Tzeng A., Kwan B.H., Opel C.F., Navaratna T., Wittrup K.D. (2015). Antigen specificity can be irrelevant to immunocytokine efficacy and biodistribution. Proc Natl Acad Sci U S A.

[89] Fallon J., Tighe R., Kradjian G. (2014). The immunocytokine NHS-IL12 as a potential cancer therapeutic. Oncotarget.

[90] Garcin G., Paul F., Staufenbiel M. (2014). High efficiency cell-specific targeting of cytokine activity. Nat Commun.

[91] Shi W., Liu N., Liu Z. (2024). Next-generation anti-PD-L1/IL-15 immunocytokine elicits superior antitumor immunity in cold tumors with minimal toxicity. Cell Rep Med.

[92] Ren Z., Zhang A., Sun Z. (2022). Selective delivery of low-affinity IL-2 to PD-1+ T cells rejuvenates antitumor immunity with reduced toxicity. J Clin Invest.

[93] Pipkin M.E., Sacks J.A., Cruz-Guilloty F., Lichtenheld M.G., Bevan M.J., Rao A. (2010). Interleukin-2 and inflammation induce distinct transcriptional programs that promote the differentiation of effector cytolytic T cells. Immunity.

[94] Kalia V., Sarkar S., Subramaniam S., Haining W.N., Smith K.A., Ahmed R. (2010). Prolonged interleukin-2Ralpha expression on virus-specific CD8+ T cells favors terminal-effector differentiation in vivo. Immunity.

[95] Mitra S., Ring A.M., Amarnath S. (2015). Interleukin-2 activity can be fine tuned with engineered receptor signaling clamps. Immunity.

[96] Mo F., Yu Z., Li P. (2021). An engineered IL-2 partial agonist promotes CD8(+) T cell stemness. Nature.

[97] Wu J., Bloch N., Chang A.Y. (2024). A PD-1-targeted, receptor-masked IL-2 immunocytokine that engages IL-2Rα strengthens T cell-mediated anti-tumor therapies. Cell Rep Med.

[98] Hsu E.J., Cao X., Moon B. (2021). A cytokine receptor-masked IL2 prodrug selectively activates tumor-infiltrating lymphocytes for potent antitumor therapy. Nat Commun.

[99] Guo J., Liang Y., Xue D. (2021). Tumor-conditional IL-15 pro-cytokine reactivates anti-tumor immunity with limited toxicity. Cell Res.

[100] Munson J.M., Shieh A.C. (2014). Interstitial fluid flow in cancer: implications for disease progression and treatment. Cancer Manag Res.

[101] Chiocca E.A., Yu J.S., Lukas R.V. (2019). Regulatable interleukin-12 gene therapy in patients with recurrent high-grade glioma: results of a phase 1 trial. Sci Transl Med.

[102] Schellhammer L., Beffinger M., Salazar U., Laman J.D., Buch T., Vom Berg J. (2023). Exit pathways of therapeutic antibodies from the brain and retention strategies. iScience.

[103] Momin N., Mehta N.K., Bennett N.R. (2019). Anchoring of intratumorally administered cytokines to collagen safely potentiates systemic cancer immunotherapy. Sci Transl Med.

[104] Momin N., Palmeri J.R., Lutz E.A. (2022). Maximizing response to intratumoral immunotherapy in mice by tuning local retention. Nat Commun.

[105] Beffinger M., Schellhammer L., Taskoparan B. (2025). FcRn-silencing of IL-12Fc prevents toxicity of local IL-12 therapy and prolongs survival in experimental glioblastoma. Nat Commun.

[106] Feng Q., Bennett Z., Grichuk A. (2024). Severely polarized extracellular acidity around tumour cells. Nat Biomed Eng.

[107] Bogdanov A., Bogdanov A., Chubenko V., Volkov N., Moiseenko F., Moiseyenko V. (2022). Tumor acidity: from hallmark of cancer to target of treatment. Front Oncol.

[108] Gaggero S., Martinez-Fabregas J., Cozzani A. (2022). IL-2 is inactivated by the acidic pH environment of tumors enabling engineering of a pH-selective mutein. Sci Immunol.

